# Evaluation of Outbreak Detection Performance Using Multi-Stream Syndromic Surveillance for Influenza-Like Illness in Rural Hubei Province, China: A Temporal Simulation Model Based on Healthcare-Seeking Behaviors

**DOI:** 10.1371/journal.pone.0112255

**Published:** 2014-11-19

**Authors:** Yunzhou Fan, Ying Wang, Hongbo Jiang, Wenwen Yang, Miao Yu, Weirong Yan, Vinod K. Diwan, Biao Xu, Hengjin Dong, Lars Palm, Shaofa Nie

**Affiliations:** 1 Department of Epidemiology and Biostatistics, School of Public Health, Tongji Medical College, Huazhong University of Science and Technology, Wuhan, China; 2 Division of Global Health (IHCAR), Department of Public Health Sciences, Karolinska Institutet, Stockholm, Sweden; 3 School of Public Health, Fudan University, Shanghai, China; 4 Institute of Public Health, Heidelberg University, Heidelberg, Germany; 5 Future Position X (FPX), Gävle, Sweden; Univ. Prince Edward Island Atlantic Veterinary College, Canada

## Abstract

**Background:**

Syndromic surveillance promotes the early detection of diseases outbreaks. Although syndromic surveillance has increased in developing countries, performance on outbreak detection, particularly in cases of multi-stream surveillance, has scarcely been evaluated in rural areas.

**Objective:**

This study introduces a temporal simulation model based on healthcare-seeking behaviors to evaluate the performance of multi-stream syndromic surveillance for influenza-like illness.

**Methods:**

Data were obtained in six towns of rural Hubei Province, China, from April 2012 to June 2013. A Susceptible-Exposed-Infectious-Recovered model generated 27 scenarios of simulated influenza A (H1N1) outbreaks, which were converted into corresponding simulated syndromic datasets through the healthcare-behaviors model. We then superimposed converted syndromic datasets onto the baselines obtained to create the testing datasets. Outbreak performance of single-stream surveillance of clinic visit, frequency of over the counter drug purchases, school absenteeism, and multi-stream surveillance of their combinations were evaluated using receiver operating characteristic curves and activity monitoring operation curves.

**Results:**

In the six towns examined, clinic visit surveillance and school absenteeism surveillance exhibited superior performances of outbreak detection than over the counter drug purchase frequency surveillance; the performance of multi-stream surveillance was preferable to signal-stream surveillance, particularly at low specificity (Sp <90%).

**Conclusions:**

The temporal simulation model based on healthcare-seeking behaviors offers an accessible method for evaluating the performance of multi-stream surveillance.

## Introduction

Syndromic surveillance collects information about health-related events prior to official diagnosis, and promotes early detection of outbreaks [1]. Such surveillance is commonplace in developed countries [2]–[5]. It is often conducted by collecting information through multiple data streams that contribute to detection effectively. Although developing countries and rural areas have attempted to create surveillance systems, their performance on outbreak detection has rarely been evaluated [6]. Of particular interest is the performance of different data streams used in surveillance system.

In outbreak detection, data streams determine whether the detection is valid and timely, and therefore worth investigating. Various data streams exist, including clinic visit, sales of over-the-counter (OTC) drugs, school/work absence, calls to help lines, environmental data, ambulance dispatch data, and others [7]–[11]. To optimize detection performance, policy makers must know which data streams are superior, and whether they are more efficient when used in parallel.

Most syndromic surveillance studies have evaluated outbreak detection performance by comparing surveillance signals with a gold standard of surveillance data, such as laboratory pathogen surveillance or conventional confirmed case surveillance [12]. In resource-poor settings, however, governments cannot afford expensive surveillance. In rural China, village clinics are equipped with simple instruments, and are unable to administer laboratory tests for disease confirmation. Furthermore, the Chinese Information System for Diseases Control and Prevention (CISDCP), a conventional routine reporting system for selected infectious diseases, cannot monitor village populations in a timely manner because the hierarchical nature of the system dictates that villages must first send cases to township staff to be recorded in the system. Thus, it is difficult to assess the performance of syndromic surveillance using limited “gold standard” data in rural areas.

An alternative approach is to use simulated data for assessment. Many studies have evaluated the performance of single-stream surveillance through simulated outbreaks. Multi-stream surveillance, however, has seldom been evaluated, because simulated outbreak data cannot be superimposed directly onto different syndromic data baselines (which represent different health-related events, such as visiting clinics, OTC drug purchasing, or absence due to illness). Simulated outbreak data must first be converted into corresponding simulated syndromic data prior to superimposition.

Because all syndromic data streams are associated with one another, we proposed a simulation method based on the healthcare-seeking behaviors that can capture the inner linkages between outbreak data and various syndromic data. Thus, a discrete probability distribution of healthcare-seeking behaviors of symptomatic individuals may be used to convert simulated outbreak data into multiple simulated syndromic data [13], [14].

## Methods

**Figure 1:**
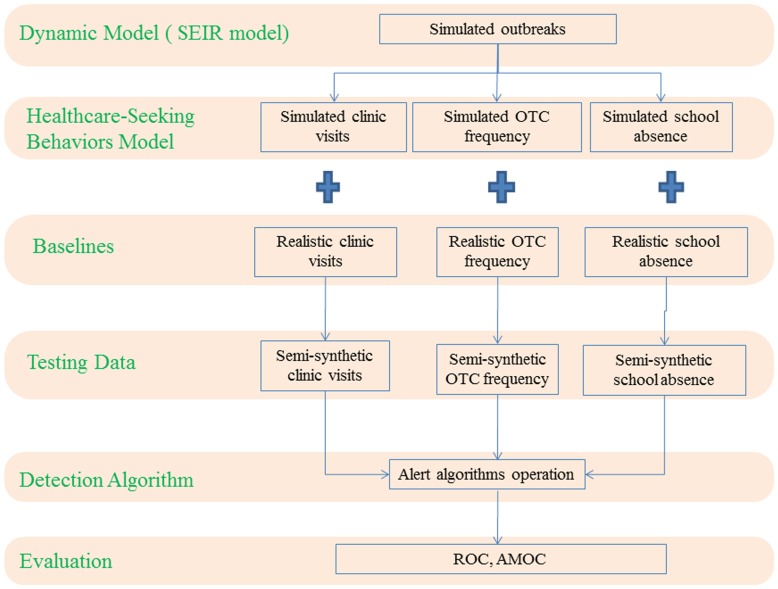
Schematic diagram of multi-stream evaluation based on healthcare-seeking behaviors model for performance on outbreak detection. First, simulated outbreak datasets are generated by the SEIR model. Second, the simulated outbreak datasets are converted into three kinds of syndromic datasets according to a discrete distribution probability of healthcare-seeking behaviors. Third, the converted syndromic datasets are superimposed onto corresponding syndromic baseline datasets to create testing datasets. Next, detection algorithms can be performed on testing datasets to detect simulated outbreaks. Last, relevant indicators can be devised to evaluate the detection performance. OTC: over-the-counter; ROC: receiver operating characteristic; SEIR: Susceptible – Exposed – Infectious – Recovered model; AMOC: activity monitoring operation curves.

We introduced an evaluation method based on the healthcare-seeking behaviors model for multi-stream syndromic surveillance (Figure 1). We designated influenza A (H1N1) as the hypothetical disease because it is highly infectious and received high attention from public health agencies worldwide since the 2009 influenza A (H1N1) pandemic.

### Simulated outbreaks model

We generated simulated influenza outbreak data based on the Susceptible – Exposed – Infectious – Recovered (SEIR) model. This model imitates four main health states in disease progression. First, individuals begin in the susceptible state (S) and progress to the exposed state (E) at rate *β* when in contact with infected individuals. Second, following an incubation period, exposed individuals move to the infectious state (I) at rate *ω*. Finally, at the end of the infectious period, infected individuals enter the recovery state (R) at rate *γ* [15], [16]. This process may be modeled using the following equations:
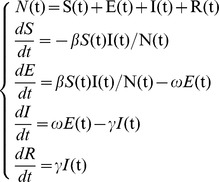
where N(t) is the total population at time t. S(t), E(t), I(t), and R(t) are the numbers of individuals at time t in each of the four states. Ratios of 1/*ω* and 1/*γ* are the mean incubation and infectious periods, respectively. *β* is the transmission rate, which reflects the diffusion intensity of a disease; it is usually measured by the reproductive number (*R_0_*), which refers to the number of secondary cases for each primary case: *R_0_*  =  *β/γ*.

To simulate influenza A (H1N1) outbreak data, we defined the values of *R_0_*, 1/*ω*, and 1/*γ*. Previous studies [15], [17], [18] estimated *R_0_* for influenza A (H1N1) to be in the range of about 1.0–3.0. Thus we defined *R_0_* at the three levels in our study as 1.5, 2.0, and 2.5. We defined 1/*ω* as 1, 2, and 3, and 1/*γ* as 3, 5, and 7, according to the natural progression of influenza A (H1N1) [19]–[22]. We then varied a single parameter and held all others fixed, resulting in 27 scenarios of simulated outbreaks (Table 1). Xu et al. reported that the Chinese population has a very low pre-existing immunity to influenza A (H1N1) virus [23]; thus, we assumed that all populations in our target sites were susceptible at the beginning of the outbreaks. In the process of simulating, we assumed equal infectiousness among populations and did not take mortality, or possible interventions (hospitalizations or treatments) into consideration.

**Table 1:** Summary of simulated outbreaks with different parameters.

Outbreak	*R_0_*	*1/ω*	*1/γ*	Total cases	Peak cases	Time to peak (d)	Duration (d)
1	1.5	3.0	3.0	34	NA	NA	46
2	1.5	3.0	5.0	41	NA	NA	57
3	1.5	3.0	7.0	46	NA	NA	74
4	1.5	2.0	3.0	43	2	17	38
5	1.5	2.0	5.0	49	2	22	48
6	1.5	2.0	7.0	53	2	38	59
7	1.5	1.0	7.0	60	2	20	50
8	1.5	1.0	3.0	53	3	19	30
9	1.5	1.0	5.0	58	3	13	41
10	2.0	3.0	3.0	78	4	23	35
11	2.0	3.0	5.0	94	4	27	44
12	2.0	3.0	7.0	104	4	33	52
13	2.0	2.0	7.0	121	5	29	44
14	2.0	2.0	3.0	98	6	17	29
15	2.0	1.0	7.0	137	6	21	37
16	2.0	1.0	5.0	131	8	21	29
17	2.5	3.0	7.0	163	8	30	41
18	2.0	2.0	5.0	112	9	27	35
19	2.5	3.0	3.0	117	9	20	26
20	2.0	1.0	3.0	121	9	20	22
21	2.5	2.0	7.0	183	10	25	40
22	2.5	3.0	5.0	140	11	26	36
23	2.5	2.0	5.0	167	11	22	29
24	2.5	2.0	3.0	146	12	17	23
25	2.5	1.0	7.0	207	13	19	29
26	2.5	1.0	5.0	196	15	16	24
27	2.5	1.0	3.0	177	20	13	17

NA: the epidemics did not manifest an obvious peak due to the low transmissibility and long incubation duration of virus.

*R_0_*: the basic reproductive number.

*1/ω*: the incubation period.

*1/γ*: the infectious period.

### Healthcare-seeking behaviors model

To superimpose the simulated outbreak data onto syndromic baselines, we needed to convert them into the corresponding syndromic data using the healthcare-seeking behaviors model. This model simulated the occurrence and timing of three types of healthcare-seeking behaviors following syndrome onset: visiting clinics, OTC drug purchasing, and school absenteeism.

**Figure 2:**
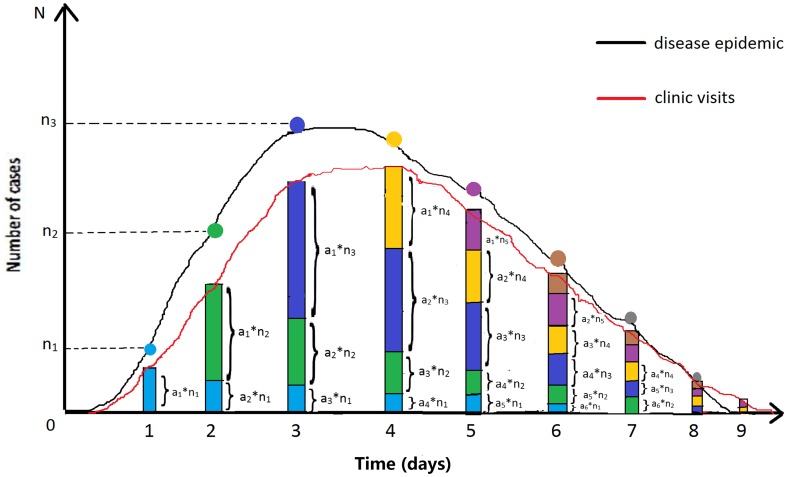
Sketch diagram of conversion principle from simulated outbreak data to syndromic data. Clinic visits data were used as an example; a_i_ is the probability of patients visiting doctors at day i; n_i_ is the number of new outbreak cases at day i. The number of new cases at day 1 is n_1_. Of these, a_1_n_1_ cases will visit doctors in clinics on that day; a_2_n_1_ cases will visit doctors on the second day; a_3_n_1_ will do so on the third day (light blue pillars), etc. On day 2, there are n_2_ new cases, of which a_1_n_2_ cases will visit doctors in clinics on that day; a_2_n_2_ cases will do so on the second day; a_3_n_2_ will do so on the third day (green pillars), etc. As a result, the sum of the visiting volumes on day 1 is a_1_n_1_; on day 2, it is a_2_n_1_ + a_1_n_2_; on day 3, it is a_3_n_1_ + a_2_n_2_ + a_1_n_3_; etc.



Figure 2 illustrates the principle of converting simulated outbreak data into syndromic data (clinic visit data are used as an example): a_i_ is the probability of patients visiting doctors at day i; n_i_ is the number of new outbreak cases at day i. As the figure shows, the number of new cases at day 1 is n_1_. Of these, a_1_n_1_ cases will visit doctors in clinics on that day; a_2_n_1_ cases will visit doctors on the second day; a_3_n_1_ will do so on the third day (light blue pillars), etc. On day 2, there are n_2_ new cases, of which a_1_n_2_ cases will visit doctors in clinics on that day; a_2_n_2_ cases will do so on the second day; a_3_n_2_ will do so on the third day (green pillars), etc. As a result, the sum of the visiting volume on day 1 is a_1_n_1_; on day 2, it is a_2_n_1_ + a_1_n_2_; on day 3, it is a_3_n_1_ + a_2_n_2_ + a_1_n_3_; etc. Thus, we formulated the relationship between outbreak data and visiting volume data as follows:

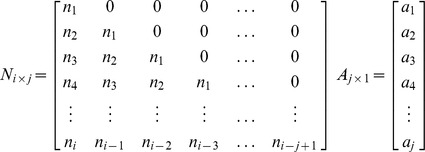


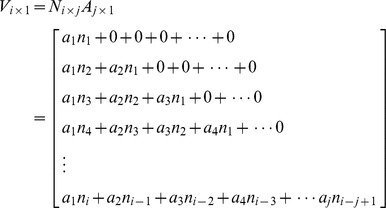
where ***N_i×j_*** is a matrix of the time-series data of new outbreak cases, and *n_i_* is the count of new cases at day *i*. The matrix ***A_j×1_*** represents the discrete probability distribution of visiting clinics for each day following the onset of symptoms, and *a_i_* is the probability of patients visiting doctors at day *i*. ***V_i×1_*** is the converted time-series data for visiting volume.

Similarly, the converted data of OTC drug purchase frequency (***O_i×1_***) can be formulated as follows:


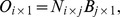
 and 
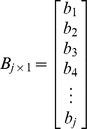
 where ***B_j×1_*** is the discrete probability distribution of OTC drug purchasing for each day following the onset of symptoms.

School absenteeism surveillance only concerns the school-aged population. We assumed the homogenous population mixing in our models, simplifying the process of disease transmission across different population. Therefore, we used the proportion of school-aged children within the population (*p*) to structure school-aged infections in simulated outbreaks. The school absenteeism data (***S_i×1_***) can be formulated as follows:


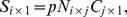
 and 
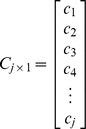
 where ***C_j×1_*** is the discrete probability distribution of absence from school for each day following the onset of symptoms, and *p* is the proportion of school-aged children within population.

Because detailed data for our target population regarding the probability of seeking care and the delay in seeking care were not available in the literature, a survey was conducted to obtain the probability and time of these behaviors. We randomly sampled 10 households in each town and 5 households in each village within the study areas. A total of 2,473 participants (including 171 school-aged children) were sampled regarding whether and when they visited doctors, purchased OTC drugs, or were absent from school, once they had symptoms of influenza-like illness (fever + cough or sore throat).

### Baseline data

The Integrated Surveillance System (ISS), the first electronic syndromic surveillance system for infectious diseases in rural China, was employed for our field experiment in six towns in Hubei province on April 1, 2012. The ISS collects daily syndromic information from three data streams: chief complaints from health clinics, medication sales from retail pharmacies, and primary school absences. Chief complaints surveillance focuses on patients' main symptoms and basic information including age, gender, home address, and visiting time. Medication sale surveillance concerns daily sales of 98 drugs. School absence surveillance concerns the daily numbers of and reasons given for absence of students from primary school. Further details of the ISS may be found in previous studies [24], [25].

We used ISS daily numbers of three syndromic data streams (clinic visit, OTC drug purchase frequency, and primary school absence) in six towns in Hubei, China (Longwang, Zhangjing, Shiqiao, Zengji, Hougang, and Xiongkou; total population in the target regions was 326,984, population density was about 326 persons/km^2^, sex ratio (male: female) was 1.03, and per capita GDP was $1,691). The ISS routinely collected data from 152 health centers (6 township hospitals, 146 village clinics), 11 township drugstores, and 26 primary schools (6 township and 20 village schools). The clinic visit (CV) data stream recorded the daily count of patients with symptoms of influenza-like illness. The OTC drug purchase frequency (OTC) data stream recorded the daily count of consumers who purchased three categories of drugs related to respiratory symptoms: antipyretics, compound cold medicine, and cough suppressants (a consumer could be recorded for only one at a time, even though he/she may have purchased multiple categories of drugs). The school absence (SA) data stream recorded the daily number of absent students claiming to experience influenza-like illness symptoms (weekends and vacations excluded, because schools are closed). We used these three data streams from April 1, 2012, to June 30, 2013 (no outbreak took place during this period), to provide realistic baseline data.

### Generation of semi-synthetic testing data

Given the complexities of real data, a semi-synthetic approach is preferable to a fully synthetic approach in assessment; the former superimposes simulated outbreak cases onto a realistic baseline and then uses this combination as testing data [13], [26]. In other words, the number of cases on the testing data at day t is the sum of the cases of simulated outbreak data and realistic baseline data at day t. Testing datasets can be calculated using the following formulas:
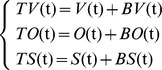
 where *TV(t)*, *TO(t)*, and *TS(t)* are the numbers of cases on testing datasets of clinic visit, OTC drug purchase frequency, and school absence at day t, respectively. *BV(t)*, *BO(t)*, and *BS(t)* are the numbers of cases on baseline datasets of each data stream at day t. *V(t)*, *O(t)*, and *S(t)* are the numbers of cases on simulated datasets of each data stream at day t.

**Figure 3:**
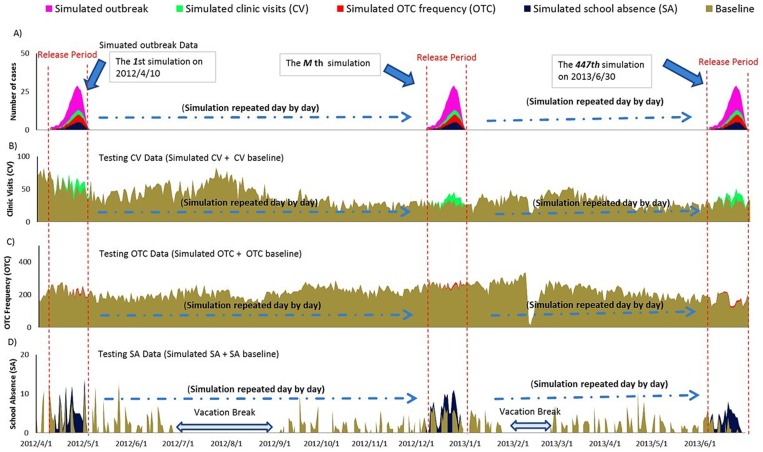
Generation of semi-synthetic testing datasets in six towns in Hubei, China, 2012/4/1–2013/6/30. A) Simulated Outbreak Data (generated by SEIR model) and converted syndromic data (generated by healthcare-seeking behaviors model); B) Testing CV Data (simulated CV + CV baseline); C) Testing OTC Frequency Data (simulated OTC + OTC baseline); D) Testing SA Data (simulated SA + SA baseline). The pink epidemic was one of the simulated outbreaks generated by the SEIR model. This could be converted into simulated clinic visits (green), simulated OTC drug purchase frequency (red), and simulated school absence (black), according to the healthcare-seeking behaviors model. The first simulated outbreak was released on 2012/4/10. Simulated syndromic data were superimposed onto corresponding baselines on the same release period (see B, C, and D). Every simulation released one outbreak to generate three testing datasets, including testing CV data, testing OTC data, and testing SA data. The simulation was repeated day by day during the whole surveillance period (2012/4/10–2013/6/30). Testing SA data on vacation breaks were defaulted as “0.” CV: clinic visits; OTC: over-the-counter; SA: school absence; SEIR: Susceptible – Exposed – Infectious – Recovered model.

Data obtained during April 1–9, 2012, provided background counts for the detection algorithm. The superimposing process began on April 10, 2012. To avoid bias due to seasonality and day-of-the-week effects, this process was repeated every day from April 10, 2012, to June 30, 2013, for each of the three realistic syndromic baselines [27]. This yielded 447 testing datasets per scenario per data stream, for a total of 36,207 (447 * 27 * 3) datasets for analysis (Figure 3).

### Detection algorithm

Because the ISS system has only been in use in rural China since 2012, we did not have long-term historical data as a background for our algorithm. Thus, a non-historical model of the Early Aberration Reporting System (EARS) was suitable for our data, which collected <2 years of background data [28]. The EARS has been increasingly used as a standard syndromic surveillance system in both the USA and China [29], [30]. The EARS models were intended to be used as the cumulative sum method (CUSUM) consisting of three algorithms—C1, C2, and C3—that show increasing sensitivities matching their intended sensitivity levels (C3 being most sensitive). The statistic of CUSUM value can be written as follows: 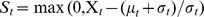
where X_t_ is the count of cases at day t, and *μ_t_* and *σ_t_* are the moving sample mean and standard deviation at baseline, respectively. The C1 baseline is obtained from the previous 7 days in closest proximity to the current day (day t-7 through day t-1). C2 uses a 7-day baseline on day t-9 through day t-3. C3 is the sum of the C2 values for the past 3 days [31].

To obtain the receiver operating characteristic (ROC) curve and activity monitoring operation curves (AMOC) [32], we set seven thresholds for each algorithm (0.1, 0.5, 1.0, 1.5, 2.0, 2.5, and 3.0). These thresholds indicate the critical CUSUM value levels when the number of current cases exceeds three deviations above the baseline mean [33].

### Surveillance strategies

We designed seven surveillance strategies according to combinations of three data streams, with three single-stream surveillance strategies: (1) Clinic Visit Only, (2) OTC drug purchase frequency Only, (3) School Absence Only; and four multi-stream surveillance strategies: (4) Clinic Visit + OTC Frequency, (5) Clinic Visit + School Absence, (6) OTC Frequency + School Absence, (7) Clinic Visit + OTC Frequency + School Absence. We defined the multi-stream signal as the earliest signal generated in any sub-data stream. The performance of different strategies was compared to allow us to judge which strategy was the best and whether multi-stream surveillance was more efficient for outbreak detection.

### Performance evaluation

The metrics used to evaluate the performance were the receiver operating characteristic (ROC) curve and activity monitoring operation curves (AMOC); these could be draw by using the 7 thresholds mentioned above. ROC curves were plotted using 1-specificity and sensitivity at each threshold. Similarly, AMOC was plotted using 1-specificity and proportional timeliness (time to detection divided by the outbreak duration). Proportional timeliness enables the direct comparison of detection timeliness across different outbreak scenarios. We calculated these indicators by averaging the detection outcome across all 12,069 (447 * 27) analysis runs in each surveillance strategy.

Sensitivity (*Se*) was defined as the number of flagged aberrations that correctly corresponded to simulated outbreaks (f), divided by the total number of simulated outbreaks (s): 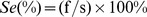.

Specificity (*Sp*) was defined as the total number of days that did not contain simulated outbreaks and remained unflagged (d), divided by the total number of days that did not contain simulated outbreaks (D): 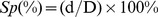.

Proportional timeliness (*Pt*) was defined as the time to detection (t, the number of days that occurred between the beginning of an outbreak and the first day the outbreak was flagged [28]), divided by the outbreak duration (T): 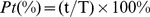.

To compare the overall performance of outbreak detection between single-stream surveillance and multi-steam surveillance, we calculated the overall sensitivity, specificity and proportional timeliness by averaging these estimates across relevant surveillance strategies:
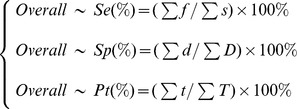



The overall estimates for single-stream surveillance were calculated by averaging strategies of (1) Clinic Visit Only, (2) OTC drug purchase frequency Only, and (3) School Absence Only. The overall estimates for multi-stream surveillance were calculated by averaging strategies of (4) Clinic Visit + OTC Frequency, (5) Clinic Visit + School Absence, (6) OTC Frequency + School Absence, (7) Clinic Visit + OTC Frequency + School Absence.

All simulations were generated using VBA programming embedded in Microsoft Excel 2007; statistical analyses were conducted using SPSS version 12.0 (SPSS Inc., Chicago, IL, USA).

### Ethics Statement

Written informed consent statements were obtained from the all relevant participants including parents of children. All participants and patients were anonymized and only aggregated data was used for data analysis. The personal identification information did not appear in the final database. The study was ethically approved by the Institutional Review Board of Tongji Medical College.

## Results

### Realistic syndromic baseline

**Figure 4:**
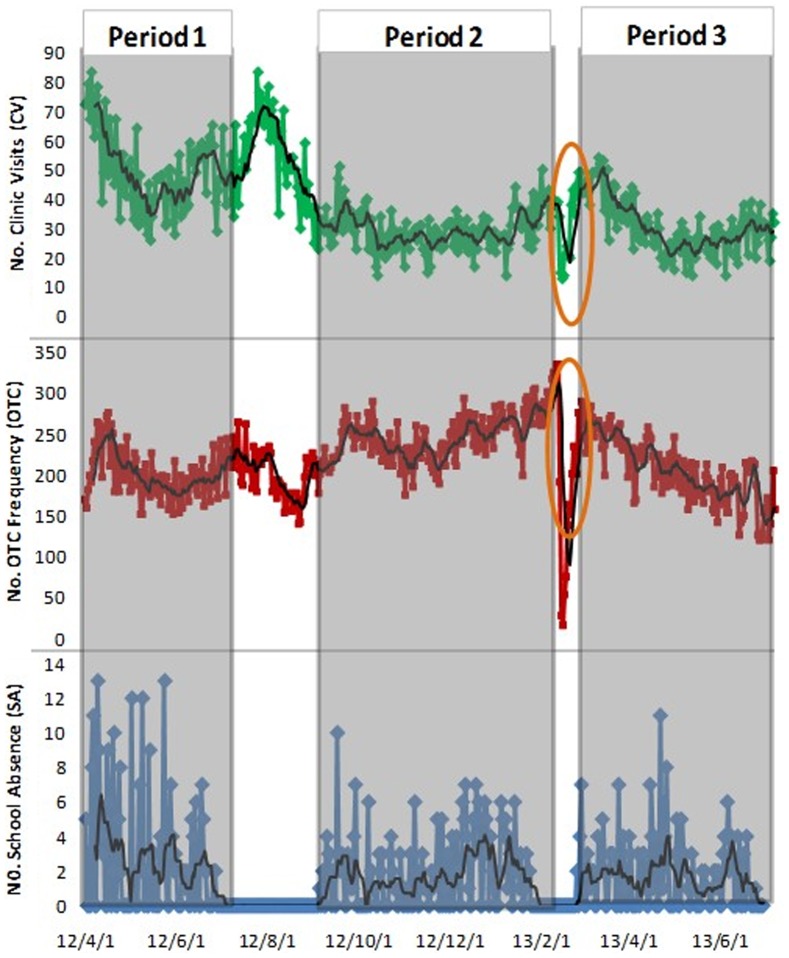
Baselines of three surveillance streams in six towns in Hubei, China, 2012/4/1–2013/6/30. Gray bars show three periods separated by vacation breaks. Circles point out dips during the Chinese New Year.

During the period lasting from April 1, 2012, to June 30, 2013, the ISS recorded 16,956 visitors due to influenza-like syndrome (37.2±14.2 per day), 98,744 respiratory syndrome-related OTC drug sales (216.5±42.6 per day), and 715 student absences due to influenza-like syndrome (2.7±2.6 per day; Table S1 in File S1). The clinic visit data stream demonstrated higher levels of activity in summer and winter, coinciding with the seasonal peaks of respiratory diseases. Similar peaks occurred in the OTC drug purchase frequency data stream, but were less obvious in summer. Dips at the end of February 2013 in both clinic visit and OTC drug purchases frequency coincided with the Chinese New Year, in line with traditional Chinese avoidance of healthcare during this period. The school absenteeism data stream did not demonstrate a noticeable pattern because of the numerous vacation breaks (Figure 4).

We also calculated Spearman's rank correlation coefficients between the different time series of data streams within three periods separated by vacation breaks (Table 2). The clinic visit stream correlated strongly with the OTC drug purchase frequency stream in all periods (maximum r = 0.79, lag  = 5∼7 days in period 1; maximum r = 0.49, lag  = 6∼7 days in period 2; and maximum r = 0.66, lag  = 0 days in period 3). The clinic visit stream correlation with the school absenteeism stream was high in period 1 (maximum r = 0.57, lag  = 2∼3 days) and period 2 (maximum r = 0.33, lag  = -3∼-4 days), but not significant in period 3. The OTC drug purchase frequency and school absenteeism streams were significantly correlated in all periods (maximum r = 0.52, lag  = -2∼-6 days in period 1; maximum r = 0.63, lag  = -12∼-13 days in period 2; and maximum r = 0.28, lag  = 3∼6 days in period 3).

**Table 2:** Cross correlation coefficient between data streams in different lags.

	Period 1			Period 2			Period 3		
Lag days	CV with OTC	CV with SA	OTC with SA	CV with OTC	CV with SA	OTC with SA	CV with OTC	CV with SA	OTC with SA
−14	−0.13	0.03	0.25*	0.22*	0.23*	0.60*	0.17	−0.03	−0.10
−13	−0.08	0.05	0.29*	0.22*	0.21*	0.63*	0.19*	−0.03	−0.05
−12	−0.03	0.07	0.33*	0.22*	0.18*	0.63*	0.23*	−0.03	0.00
−11	0.02	0.08	0.37*	0.19*	0.16	0.60*	0.26*	−0.02	0.04
−10	0.07	0.08	0.42*	0.15	0.15	0.56*	0.30*	−0.01	0.09
−9	0.12	0.10	0.47*	0.11	0.16	0.51*	0.35*	0.01	0.13
−8	0.18	0.12	0.49*	0.07	0.18*	0.48*	0.40*	0.04	0.16
−7	0.23*	0.12	0.50*	0.05	0.22*	0.45*	0.44*	0.05	0.17
−6	0.29*	0.13	0.52*	0.04	0.26*	0.42*	0.49*	0.05	0.17
−5	0.35*	0.15	0.52*	0.04	0.31*	0.41*	0.53*	0.04	0.17
−4	0.40*	0.20	0.52*	0.07	0.33*	0.43*	0.56*	0.03	0.17
−3	0.45*	0.26*	0.52*	0.11	0.33*	0.44*	0.59*	0.01	0.17
−2	0.51*	0.31*	0.52*	0.15	0.32*	0.46*	0.62*	−0.02	0.18*
−1	0.56*	0.36*	0.51*	0.20*	0.29*	0.46*	0.64*	−0.05	0.21*
0	0.62*	0.43*	0.49*	0.26*	0.25*	0.45*	0.66*	−0.06	0.24*
1	0.67*	0.50*	0.48*	0.30*	0.20*	0.44*	0.65*	−0.08	0.26*
2	0.73*	0.57*	0.46*	0.36*	0.15	0.43*	0.64*	−0.08	0.27*
3	0.76*	0.57*	0.43*	0.39*	0.12	0.40*	0.62*	−0.09	0.28*
4	0.78*	0.52*	0.39*	0.44*	0.09	0.37*	0.61*	−0.08	0.28*
5	0.79*	0.46*	0.37*	0.47*	0.05	0.35*	0.61*	−0.05	0.28*
6	0.79*	0.41*	0.35*	0.49*	0.01	0.34*	0.60*	−0.02	0.28*
7	0.79*	0.35*	0.31*	0.49*	−0.03	0.34*	0.60*	0.00	0.26*
8	0.78*	0.27*	0.27*	0.46*	−0.06	0.34*	0.61*	0.02	0.24*
9	0.75*	0.21	0.23*	0.41*	−0.08	0.34*	0.61*	0.03	0.22*
10	0.71*	0.17	0.18	0.37*	−0.10	0.33*	0.61*	0.05	0.19*
11	0.68*	0.14	0.12	0.30*	−0.13	0.33*	0.62*	0.06	0.17
12	0.63*	0.13	0.06	0.24*	−0.11	0.33*	0.61*	0.06	0.16
13	0.56*	0.13	0.02	0.18*	−0.09	0.31*	0.60*	0.06	0.16
14	0.52*	0.11	−0.01	0.14	−0.07	0.28*	0.60*	0.06	0.16

* Cross correlation coefficient is significant (*P*≤0.05).

CV: clinic visits; OTC: over-the-counter; SA: school absence.

### Generation of simulated outbreaks

Using the SEIR model, 27 scenarios of simulated outbreak were generated using different combinations of parameters (Table 1 and Table S2 in File S1). The number of infected individuals varied from 34 to 207. Outbreak 27 was the strongest, with a maximum of 20 cases occurring on peak day. The lowest three outbreaks were 1, 2, and 3 lasting for a long time without peaks; these were also more likely to be sporadic outbreaks. Total cases and peak cases rose in accordance with an increased value of R_0_, whereas increasing R_0_ decreased peak time and total duration. A decrease in the value of 1/ω (i.e., a decrease in the incubation period) raised the number of total cases and peak cases, and decreased peak time and duration. In contrast, raising the value of 1/γ (e.g., an increase in the infectious period) increased the number of total cases and duration, but did not significantly influence peak cases and peak time.

### Healthcare-seeking behaviors pattern

The healthcare-seeking behavior questionnaires targeting an influenza-like syndrome were completed and returned with an overall response rate of 75.7% (n = 1,873 of 2,473; 53.0% male; 40.2 (17.9) years of age; 6.9% primary-school age population). Table 3 shows the discrete probability distribution of healthcare-seeking behaviors following the onset of syndrome. Of the total population, 51.3% (960/1,873) participants replied that they would visit a doctor, and 39.8% (746/1,873) would purchase drugs. In the primary school-age population, 25.4% (33/130) would miss school. Using these parameters, we converted 27 simulated outbreak datasets into 81 relevant syndromic datasets (Table S3 in File S1).

**Table 3:** Probability distribution of healthcare-seeking behaviors of residents after onset of influenza-like syndrome.

Time to seek healthcare	Visiting doctors		Purchasing drugs		Absence from schools*	
	N	a_i_(%)	N	b_i_(%)	N	c_i_(%)
1 day	537	28.7	428	22.9	20	15.4
2 days	343	18.3	274	14.6	8	6.2
3 days	70	3.7	38	2.0	5	3.8
4 days	8	0.4	6	0.3	0	0.0
5 days	1	0.1	0	0.0	0	0.0
6 days	1	0.1	0	0.0	0	0.0
No behavior	913	48.7	1127	60.2	97	74.6
Total	1873	100.0	1873	100.0	130	100.0

*Population absent from schools refers to school-aged children (5–14 years old). The a_i_, b_i_, and c_i_ values are proportions of relevant healthcare behaviors at day i, which are used as parameters in ***A_j×1_*** *,* ***B_j×1_*** *, and* ***C_j×1_*** in the healthcare-seeking behaviors model.

### Validity and timeliness of syndromic surveillance

**Figure 5:**
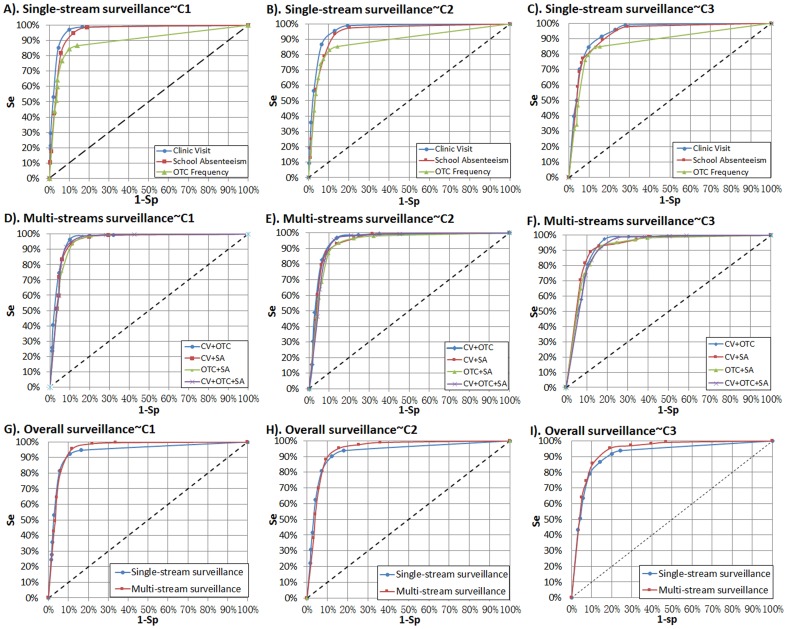
Comparison of validities for all surveillance strategies using EARS ∼ 3Cs algorithms. CV: clinic visit surveillance; EARS: the Early Aberration Reporting System; OTC: over-the-counter frequency surveillance; SA: school absence surveillance. Overall single-stream surveillance contains strategies of CV, OTC, and SA; overall multi-stream surveillance contains strategies of CV + OTC, CV + SA, OTC + SA, and CV + OTC + SA.

Figure 5 shows the ROCs of all surveillance strategies. In single-stream surveillance strategies (Figure 5-A, B, C), the clinic visit stream manifested the highest validity, while the OTC drug purchase frequency stream exhibited the lowest validity for all algorithms. In multi-stream surveillance strategies (Figure 5-D, E, F), all strategies exhibited similar performance for outbreak detection. We also plotted the ROCs of overall single-stream and multi-stream surveillance (Figure 5-G, H, I). According to the comparison results, overall multi-stream surveillance had superior sensitivity to overall single-stream surveillance, especially when specificities were below 90% (namely 1-Sp>10%).

**Figure 6:**
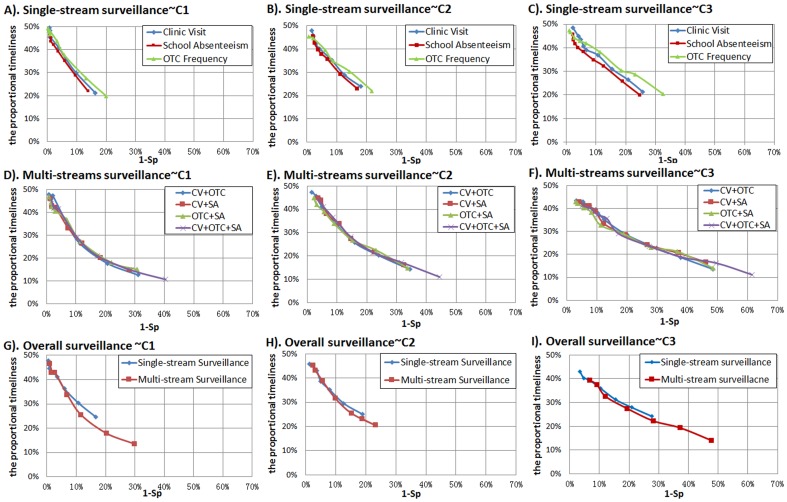
Comparison of timeliness for all surveillance strategies using EARS ∼ 3Cs algorithm. CV: clinic visit surveillance; EARS: the Early Aberration Reporting System; OTC: over-the-counter frequency surveillance; SA: school absence surveillance. Overall single-stream surveillance contains strategies of CV, OTC, and SA; overall multi-stream surveillance contains strategies of CV + OTC, CV + SA, OTC + SA, and CV + OTC + SA.

Figure 6 shows the AMOC curves of all surveillance strategies. In single-stream surveillance (Figure 6-A, B, C), the school absenteeism stream had a slightly superior timeliness than the other two data streams for all algorithms; this, however, was not obvious at a higher level of specificity. In multi-stream surveillance (Figure 6-D, E, F), all strategies exhibited similar timeliness of outbreak detection. By comparing overall single-stream and multi-stream surveillance (Figure 6-G, H, I), we found that overall multi–stream surveillance had superior timeliness to overall single-stream surveillance when specificities were below 90% (namely 1-Sp>10%). However, at a high level of specificity, there was little difference in timeliness between single-stream and multi-stream surveillance for all algorithms.

Meanwhile, comparing the positions of ROC and AMOC curves between different detection algorithms, we found that the EARS ∼ C1 model exhibited the best validity (ROC curves of C1 were closest to the top left origin) and timeliness (AMOC curves of C1 were closest to the bottom left origin), while the EARS ∼ C3 model was slightly inferior to the other two algorithms in our study.

## Discussion

We explored the performance of multi-stream syndromic surveillance on outbreak detection in rural Hubei, China by using simulation influenza A (H1N1) outbreaks based on the healthcare-seeking behaviors model. Although several other studies have previously evaluated the performance of syndromic surveillance through simulation methods, most had done so by evaluating a single data stream [2], [14], [27], [31]. One study considered concurrent surveillance of two data streams [34]; however, only a simple and fixed probability of healthcare-seeking was used, and did not factor in the time individuals sought care. In fact, all syndromic data streams were associated with each other. A simulation based on the healthcare-seeking behaviors model, which assessed individuals' healthcare-seeking behavior patterns following the onset of symptoms, is a useful framework for simulating associated syndromic datasets.

Results from our study areas showed that clinic visit surveillance exhibited the most favorable validity, similar to findings of previous studies [35], [36]. The clinic visit data stream, which collects individual medical details including demographic characteristics and chief complaints, makes it easy to screen out visitors using precise symptoms related to specific diseases. Detail individual chief complaints can help to exclude those visitors who did not have symptom related to the target diseases. This decreases the non-specific noise bias of baseline datasets. Consequently, the fluctuations of visit volume data in clinics could largely be influenced by outbreaks, and allow for fine detection. When compared to the CV stream, the SA stream collected rough individual information of absence reasons that was obtained from patients, and the OTC stream could never collect individual information about reasons for medicine purchases due privacy concerns.

Like some previous studies [12], [37], we also found that SA showed a satisfactory performance of outbreak detection. Primary school-aged students who gather regularly in a relatively closed and crowded environment, allow for diseases to spread easily. Therefore, school absenteeism surveillance may be more sensitive to contagious diseases. Additionally, although only 25.4% of primary school-aged students would miss school after the onset of an influenza-like syndrome (Table 3), it still led to a drastic fluctuation compared to the relatively low baseline data (Figure 3-D), so that outbreaks could still be detected effectively. There are, however, some disadvantages to school absenteeism surveillance, such as limited coverage (only school-aged children), and intermittent surveillance due to schools being closed on weekends, and vacations.

In our study areas, the OTC drug purchase frequency surveillance exhibited inferior performance of outbreak detection, when compared to clinical visit and school absenteeism surveillance. We surmise that this occurred due to the fact that drug sale information is less specific to diseases (not all consumers buy drugs for illnesses; and drug sale records contain no individual medical information; moreover drugs may be preserved during a long period of time and to be used later). Additionally, the OTC drug purchase frequency baseline (an average of 216 persons per day) was much higher, so that the extra sales volume resulting from outbreaks was not apparent (Figure 3-C). Indeed, fluctuation of surveillance data can generally be influenced by the baseline. Outbreak is easier to detect when the incidence and variation of the baseline count are low relative to outbreak cases. Researchers have reported that outbreaks with a magnitude of less than 10% of the baseline are difficult to detect when operating at a high specificity [2]. Therefore, detection performance of OTC drug purchase frequency surveillance was discounted by a dilution effect resulting from a large degree of non-specific noises in the baseline data.

Some modifications of OTC drug purchase frequency surveillance in SSI are worth performing in the future, such as collecting individual medical information as soon as possible and reducing non-specific baseline counts through a more refined classification of drug categories.

Our study also showed that multi–stream syndromic surveillance could improve the performance of outbreak detection at a low level of specificity; however, this improvement was not manifested when the specificity level was above 90%. In fact, multi–stream syndromic surveillance seems to improve detection performance through the collection of a greater amount of pre-clinical information, but does so at the cost of non-specific signals. The more data streams are used, the more non-specific signals will be captured; this may be a possible reason for the multi-stream surveillance's superior performance of outbreak detection at a lower specificity. In practice, researchers usually increase the algorithm threshold to get a high specificity for outbreak detection. The outbreaks that can be detected by a higher threshold, however, usually have a stronger intensity, and a stronger outbreak can, itself, give rise to drastic fluctuations in relevant syndromic surveillance data streams. Therefore, in general, severe outbreaks can be easily detected by both single-stream and multi-stream surveillance. This bias inherent to stronger outbreaks that can be detected at a high threshold may partly explain the similar outbreak detection performance exhibited by both single-stream and multi-stream surveillance at a high specificity.

Like previous studies [28], [33], our results also found that C1 had the best timeliness of detection due to the fact that it used data from 7 previous days of closest proximity to the current day (day t-7 through day t-1) as baseline, while C2 and C3 used data from day t-9 through day t-2 as baseline. Additionally, we found C1 was also the superior model for validity of outbreak detection in our study sites, although, the optimal model validity among C1, C2, and C3 may vary across the size, distribution, and duration of outbreaks [27].

Although outbreak simulation methods allowed for greater flexibility and evaluated the performance of aberration detection quantitatively, generalization from simulated outbreaks to real outbreaks was far from straightforward [2]. Some limitations to our simulation methods and assumptions should be addressed.

First, the dynamic model used to simulate the infectious disease transmission in our study belongs to a deterministic model, which is defined via a system of ordinary differential equations. An attractive feature of this deterministic model is that it describes, in a straightforward manner, how the number of infections evolves through time. Nevertheless, disease transmission in real world is stochastic and complex. Therefore, stochastic models may be more appropriate than deterministic models with regard to fitting models to data [38]. Some studies have reported relevant methods, such as specifying probability distributions to the incubation and infectious periods [16], and estimating the essential parameters through the Markov chain Monte Carlo (MCMC) method [39] or the sequential Bayesian method [40].

Second, the host population was homogeneously mixed, meaning that simulated outbreaks did not account for the heterogeneous nature of human contact. All individuals were equally likely to come into contact with every other individual. This ignored the actual diversity of diseases transmission across different populations, such as transmissions in families, schools, or social circles. In fact, in the healthcare-seeking behaviors model, we also used a simple ‘p’ (the proportion of school-aged population) to structure the simulated school-aged infections. This likely underestimated the number of simulated school-aged infections by ignoring the vulnerability of children to influenza, as well as the fact that school children tend to spread diseases within the school environment [41].

Third, we assumed that the entire population was susceptible at the beginning of the simulation. On one hand, we thought this was reasonable for a very low pre-existing immunity to influenza A (H1N1) virus in Chinese population [23]; on the other hand, a simulation based on an entirely susceptible population could reflect emerging infectious diseases or bioterrorism, which are the likely focus of syndromic surveillance.

Fourth, the simulated population was static. Natural population change (birth rate and death rate) was ignored, because disease outbreaks did not last for a longer period than usual.

Fifth, recall bias may existed in the questionnaire survey of healthcare-seeking behaviors. Information about healthcare-seeking behaviors was obtained from the memory of local residents by asking them to remember how they sought health care during their last influenza-like illness. For this reason, we did not take syndrome severity stratification into consideration, since syndrome severity could be biased largely towards inaccurate memories and subjective judgments of residents who received the survey.

Sixth, for the parameters of the healthcare-seeking behaviors model that were obtained from the local residents' survey, the results of our study are only suitable to the six towns in rural Hubei, China. Although we could not extend them to other regions or populations, our evaluation methods based on the healthcare-seeking behaviors model may be applied anywhere.

Finally, this paper was merely a preliminary exploration on the evaluation methods based on the healthcare-seeking behavior model, assessing three data streams in rural Hubei, China. Other data streams remain potential options for assessment using this method as long as they provide information on relevant parameters of healthcare-seeking behaviors, such as nurse hotline calls or workplace absenteeism.

Overall, outbreak simulation based on the healthcare-seeking behaviors model offers a method for evaluating detection performance of multi-stream syndromic surveillance. In the six towns within our study areas, clinic visit surveillance and school absenteeism surveillance exhibited a satisfactory performance on outbreak detection; multi-stream surveillance yielded superior validity and timeliness than single-stream surveillance at low specificity (Sp <90%). We aim to explore other potential factors, such as stratification by age, detection scales, and outbreak intensity in future research.

## Supporting Information

File S1 Table S1, Baseline data of syndromic surveillance. Table S2, The 27 scenarios of simulated outbreaks generated by the SEIR model. Table S3, Converted syndromic datasets generated by the healthcare-seeking behavior model.
(XLS)
